# Supplementary material for: Yiai Fuzheng decoction inhibits triple-negative breast cancer by remodeling the immune microenvironment
Source: Front Immunol. 2025 Sep 30;16:1615631. doi: 10.3389/fimmu.2025.1615631 (PMC12518410; doi:10.3389/fimmu.2025.1615631)
Supplement: Supplementary file 2 [file DataSheet1.zip › Supplementary File 2/Treatment/D_vs_H/07_Pathway_Analysis/Pathway_Analysis/Sig_Pathways_Name/beta-Alanine metabolism.pdf]

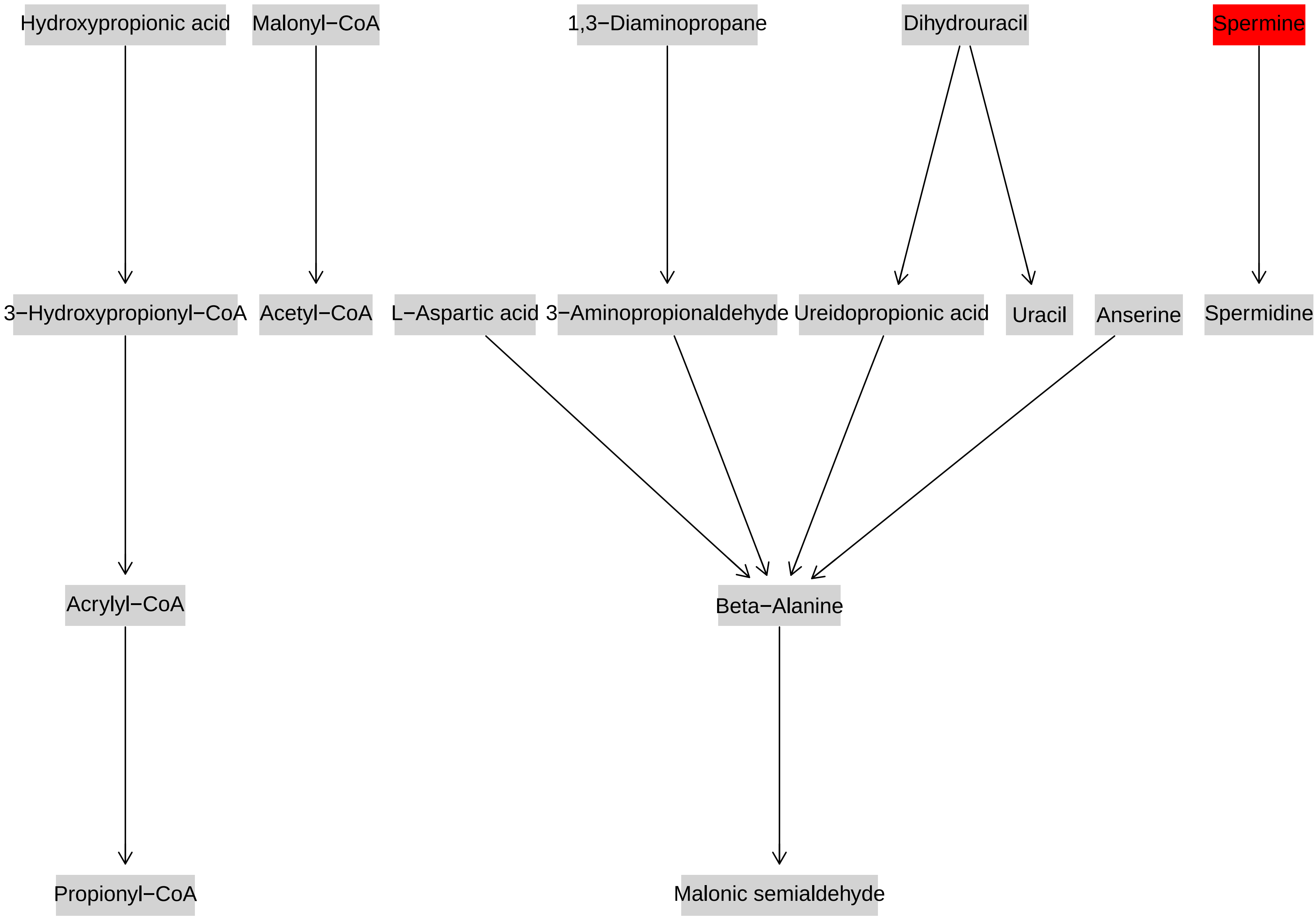
Hydroxypropionic acid
Malonyl−CoA
1,3−Diaminopropane
Dihydrouracil
Spermine
3−Hydroxypropionyl−CoA
Acetyl−CoA
L−Aspartic acid
3−Aminopropionaldehyde
Ureidopropionic acid
Uracil
Anserine
Spermidine
Acrylyl−CoA
Beta−Alanine
Propionyl−CoA
Malonic semialdehyde
